# Distinct types of eigenvector localization in networks

**DOI:** 10.1038/srep18847

**Published:** 2016-01-12

**Authors:** Romualdo Pastor-Satorras, Claudio Castellano

**Affiliations:** 1 Departament de Física, Universitat Politècnica de Catalunya, Campus Nord B4, 08034 Barcelona, Spain; 2 Istituto dei Sistemi Complessi (ISC-CNR), Via dei Taurini 19, I-00185 Roma, Italy; 3 Dipartimento di Fisica, “Sapienza” Università di Roma, P.le A. Moro 2, I-00185 Roma, Italy

## Abstract

The spectral properties of the adjacency matrix provide a trove of information about the structure and function of complex networks. In particular, the largest eigenvalue and its associated principal eigenvector are crucial in the understanding of nodes’ centrality and the unfolding of dynamical processes. Here we show that two distinct types of localization of the principal eigenvector may occur in heterogeneous networks. For synthetic networks with degree distribution *P*(*q*) ~ *q*^−*γ*^, localization occurs on the largest hub if *γ* > 5/2; for *γ* < 5/2 a new type of localization arises on a mesoscopic subgraph associated with the shell with the largest index in the K-core decomposition. Similar evidence for the existence of distinct localization modes is found in the analysis of real-world networks. Our results open a new perspective on dynamical processes on networks and on a recently proposed alternative measure of node centrality based on the non-backtracking matrix.

An issue of paramount significance regarding the analysis of networked systems is the identification of the most important (or *central*) vertices^1^. The *centrality* of a vertex may stem from the number of different vertices that can be reached from it, from the role it plays in the communication between different parts of the network, or from how closely knit its neighborhood is. Following these approaches, different centrality measures have been defined and exploited, such as degree centrality, betweenness centrality^2^, or the *K*-core index and associated *K*-core decomposition^3^. Among those definitions, one of the most relevant is based on the intuitive notion that nodes are central when they are connected to other central nodes. This concept is mathematically encoded in the *eigenvector centrality*^4^ (EC) of node *i*, defined as the component *f*_*i*_ of the principal eigenvector (PEV) **f** associated with the largest eigenvalue Λ_1_ of the adjacency matrix *A*_*ij*_. EC is the simplest of a family of centralities based on the spectral properties of the adjacency matrix including, among others, Katz’s centrality^5^ and PageRank^6^.

Apart from providing relevant information about the network structure^1^, the PEV and associated largest eigenvalue play a fundamental role in the theoretical understanding of the behavior of dynamical processes, such as synchronization^7^ and spreading^8^,^9^, mediated by complex topologies. Considerable effort has thus been devoted in recent years to the study of the spectral properties of heterogeneous networks^10^,^11^,^12^,^13^. In this framework, Goltsev *et al.*^14^ (see also^15^,^16^) have considered the *localization* of the PEV, i.e., whether its normalization weight is concentrated on a small subset of nodes or not. More in detail, let us consider an ensemble of networks of size *N*, with a PEV *f*_*i*_ normalized as a standard Euclidean vector, i.e. 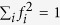. An eigenvector is *localized* on a subset *V* of size *N*_*V*_ if a finite fraction of the normalization weight is concentrated on *V* 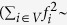 (1)) despite the fact that *V* is not extensive, i.e., *N*_*V*_ is not proportional to *N*. This includes the case of localization on a finite set of nodes (i.e. *N*_*V*_ independent of *N*, *N*_*V*_ = 1 in the extreme case of localization on a single node), but also the case of localization on a mesoscopic subset of nodes for which *N*_*V*_ ∼ *N*^*β*^ with *β* < 1. Otherwise, the eigenvector is instead *delocalized*, and a finite fraction of the nodes *N*_*V*_ ~ *N* contribute to the normalization weight, implying that their components are *f*_*i*_ ~ *N*^−1/2^.

In this context, Goltsev *et al.*^14^ study the localization in power-law distributed networks, with a degree distribution scaling as *P*(*q*) ~ *q*^−*γ*^, for which the leading eigenvalue Λ_1_ is essentially given by the maximum between 〈*q*^2^〉/〈*q*〉 and 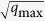, where *q*_max_ is the largest degree in the network^9^,^11^. For *γ* > 5/2, where 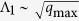, Goltsev *et al.*^14^ find that the PEV becomes localized around the hub with degree *q*_max_^14^. On the other hand, they argue that, for *γ* < 5/2, when Λ_1_ ~ 〈*q*^2^〉/〈*q*〉, the PEV is delocalized. These observations are relevant in different contexts. Firstly, they point out a weakness of EC as a measure of centrality for heterogeneous power-law networks (*γ* > 5/2), because of the exceedingly large role of the largest hub^16^. On the other hand, in the so-called quenched mean-field approach^9^,^17^ to epidemic spreading on networks, the density of infected individuals in the steady state can be related to the properties of the PEV^14^. The localization occurring for large *γ* implies that the density of infected individuals in the steady state in those processes might not be an extensive quantity, casting doubts on the validity of this theoretical approach and on the actual onset of the endemic infected state.

Here we show that the localization properties of the adjacency matrix PEV for heterogeneous (power-law distributed) networks are described by a picture much more complex than previously believed. In fact, we provide strong numerical evidence that the EC in heterogeneous networks never achieves full delocalization. In the case of uncorrelated synthetic networks with a power-law degree distribution, we obtain, by means of a finite-size scaling analysis, that for mild levels of heterogeneity (with *γ* > 5/2), the EC is strongly localized on the hubs, as previously argued. For high heterogeneity (*γ* < 5/2), however, we point out that the EC, as measured by the components of the PEV, is highly correlated with the corresponding node’s degree. This strong correlation results in an effective localization on a mesoscopic subgraph, that can be identified as the shell with the largest index in the *K*-core decomposition of the network^3^. The paper of Goltsev *et al.*^14^ is perfectly correct for what concerns the case *γ* > 5/2 but, by only considering the possibility of localization on a finite set of nodes, could not detect the mesoscopic localization occurring for *γ* < 5/2. In order to overcome the localization effects intrinsic of the EC, a new centrality measure, based on the largest eigenvalue of the Hashimoto, or non-backtracking, matrix, has been recently proposed^16^. We observe that this new centrality is not completely free from localization effects. Thus, while it almost coincides with the EC for *γ* < 5/2, and for *γ* > 5/2 it avoids the extreme localization around the hubs shown by the EC, it is still localized in this case in some mesoscopic subset of nodes, whose characterization calls for further research. The extension of our analysis to the case of real world networks is hampered by the fact that usually only one network instance is available, which prevents performing a finite-size scaling study. Nevertheless, we numerically argue that also for real networks a twofold scenario holds, in which the PEV is either localized on the hubs, or effectively localized on the maximum *K*-core of the network.

## Results

### Eigenvector localization and the inverse participation ratio

A full characterization of an undirected network of size *N* is given by its adjacency matrix^1^ **A**, whose elements take the value *A*_*ij*_ = 1 if nodes *i* and *j* are connected by an edge, and value *A*_*ij*_ = 0 otherwise. The spectral properties of the adjacency matrix are defined by the set of eigenvalues Λ_*i*_, and associated eigenvectors **f**(Λ_*i*_), *i* = 1, …, *N*, defined by


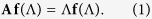


Since the adjacency matrix is symmetric all its eigenvalues are real. The largest of those eigenvalues Λ_1_, is associated with the principal eigenvector (PEV) which we denote simply by **f**.

The concept of the localization of the PEV **f** translates in determining whether the value of its normalized components is evenly distributed among all nodes in the network, or either it attains a large value on some subset, and is much smaller in all the rest. While this concept is quite easy to grasp, assessing it in a single network instance is a delicate issue because any quantitative definition involves some degree of arbitrariness. The task becomes however straightforward when ensembles of networks of different size can be generated. In such a case, the localization of the eigenvector **f** associated with the eigenvalue Λ can be precisely assessed by computing the inverse participation ratio (IPR), defined as^14^,^16^,


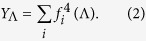


In the absence of any knowledge about the localization support, it is possible to determine whether an eigenvector is localized (on some subset in the network) by studying its inverse participation ratio, as a function of the system size *N* and fitting its behavior to a power-law decay of the form


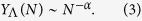


If the eigenvector is delocalized, i.e. for *f*_*i*_ ~ *N*^−1/2^, the exponent *α* is equal to 1. An exponent *α* < 1 is evidence that some form of localization is taking place. In the case of extreme localization on a single node, or on a set of nodes with size *N*_*V*_ independent of the network size *N*, the corresponding components of the PEV are finite and this implies 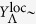  (1), i.e., *α* = 0 for *N* → ∞. Finally, if localization takes place over a subextensive set of nodes of size *N*_*V*_ ~ *N*^*β*^, we expect


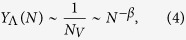


leading to a decay exponent *α* = *β*.

### Eigenvector localization in synthetic networks

**Figure 1:**
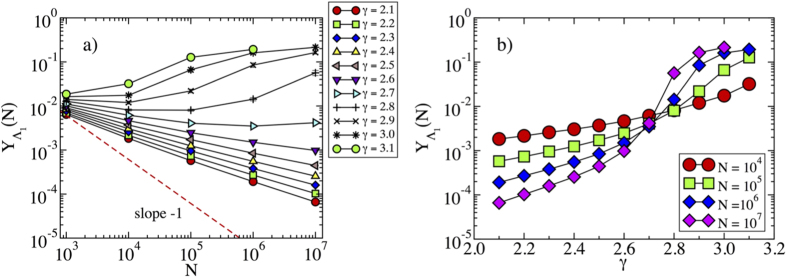
(**a**) Inverse participation ratio as a function of the network size for the adjacency matrix of synthetic networks with different degree exponent *γ*. For large *γ*, the IPR tends to saturate to a constant value for sufficiently large value of *N*. For *γ* < 5/2, on the other hand, the behavior of the IPR can be fitted to 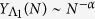, with *α* < 1/2. The dashed line represents a power-law behavior ~*N*^−1^, corresponding to a delocalized IPR. (**b**) Inverse participation ratio as a function of the degree exponent *γ* for different network sizes *N*. The plot confirms the presence of transition in the behavior of the IPR, located in the vicinity of *γ* = 5/2.

We study the localization properties of the PEV computed for synthetic power-law distributed networks of growing size, generated using the uncorrelated configuration model (UCM)^18^, a modification of the standard configuration model^19^,^20^ designed to avoid degree correlations^21^. In order to explore the presence or absence of localization, we analyze the scaling of *Y*_Λ_(*N*) as function of *N* as discussed above. In Fig. 1(a) we apply this finite-size scaling analysis to synthetic networks with different values of *γ*. In this and the following figures, statistical averages are performed over at least 100 different network samples. Error bars are usually smaller than the symbol sizes. In the case of large *γ* we observe an IPR tending to a constant for large *N*, confirming the localization on the hubs predicted by refs. 14,15. The situation is however surprisingly different for *γ* < 5/2. Thus, while according to Goltsev *et al.*^14^, we should expect a delocalized PEV and an IPR decreasing as *N*^−*α*^ with *α* = 1, we observe instead power-law decays with *N*, with effective exponents *α* always smaller than 1/2. The change of behavior of the IPR can be further confirmed in Fig. 1(b), where we plot the IPR as a function of the degree exponent *γ*, for different values of *N*. While it is clear that for *γ* ≥ 2.7 the IPR tends to a constant asymptotically, slow crossover effects do not allow to draw firm conclusions based on numerics about the precise value of *γ* for which the behavior changes. However, since the dependence of the largest eigenvalue on *N* changes for *γ* = 5/2^11^ we expect the transition to take place exactly at *γ* = 5/2: simulation results are perfectly compatible with this result.

The behavior at *γ* < 5/2 can be understood mathematically by observing that the largest eigenvalue in this regime, Λ_1_ = 〈*q*^2^〉/〈*q*〉^11^, coincides with the largest eigenvalue of the adjacency matrix in the annealed network approximation. The annealed network approximation^22^,^23^ consists in replacing the actual, fixed, adjacency matrix by an average performed over degree classes, taking the form


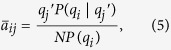


where *P*(*q*|*q*′) is the conditional probability that a link from a node of degree *q*′ points to a node of degree *q*^24^. For degree uncorrelated networks, with *P*(*q*|*q*′) = *qP*(*q*)/〈*q*〉^25^, we obtain an averaged adjacency matrix


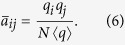


The matrix 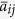 is semi-positive definite and therefore all its eigenvalues are non-negative^26^. Then considering that 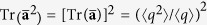, where Tr(⋅) is the trace operator, we have that 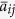 has a unique non-zero eigenvalue Λ_an_ = 〈*q*^2^〉/〈*q*〉, with associated principal eigenvector 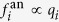. Applying the normalization condition 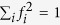, we obtain the normalized form


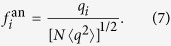


**Figure 2:**
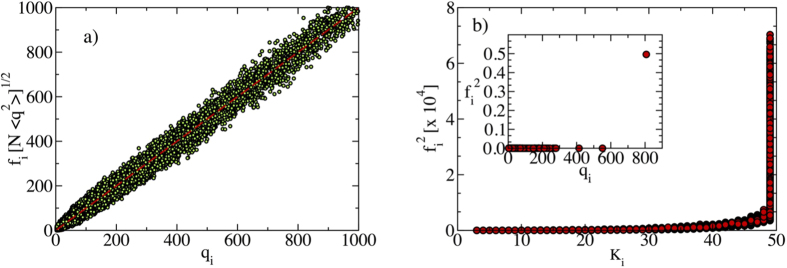
(**a**) Rescaled scatter plot of *f*_*i*_[*N*〈*q*^2^〉]^1/2^ as a function of *q*_*i*_ for a synthetic network with *γ* = 2.1 and size *N* = 10^6^. Data fits the expectation for the PEV in the annealed network approximation, Eq. (7), with only small fluctuations. (**b**, main) Scatter plot of the squared PEV components as a function of the *K*-core index for the adjacency matrix of a power-law synthetic network with *γ* = 2.1 and size *N* = 10^6^. (**b**, inset) Scatter plot of the squared PEV components as a function of the degree *q*_*i*_ in a synthetic network with *γ* = 3.5 and size *N* = 10^6^.

Inserting the expression of 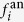 into Eq. (2) yields


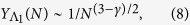


that is, a decay with an exponent smaller than 1/2, in agreement with the results in Fig. 1(b). Figure 2(a) confirms that also quenched synthetic networks have PEV components proportional in average to the degree. Notice that Eq. (8) is approximately true only in quenched networks for *γ* < 5/2, since the condition leading to it, Eq. (7) fails at *γ* > 5/2, see Fig. 2(b, inset).

A more physical interpretation of the particular distribution of the PEV in power-law networks with *γ* < 5/2, is that the PEV becomes *effectively* localized on the max(imum) *K*-core of the network, defined as the set of nodes with the largest core index *K*_*M*_ in a *K*-core decomposition^3^,^27^. The *K*-core decomposition is an iterative procedure to classify vertices of a network in layers of increasing density of connections. Starting with the full graph, one removes the vertices with degree *q* = 1, i.e. with only one connection. This procedure is repeated until only nodes with degree *q* ≥ 2 are left. The removed nodes constitute the *K* = 1-shell and those remaining compose the *K* = 2-core. At the next step all vertices with degree *q* = 2 are removed, thus leaving the *K* = 3-core. The procedure is repeated iteratively. The maximum *K*-core (of index *K*_*M*_) is the set of vertices such that one more iteration of the procedure removes all of them. The line of argument leading to this interpretation stems from combining the results of ref. 14, in which it is proposed that, in epidemic spreading in complex networks^28^, infection activity is localized on the PEV, with the observations in ref. 29, in which the maximum *K*-core is identified as a subset of nodes sustaining epidemic activity for *γ* < 5/2.

**Figure 3:**
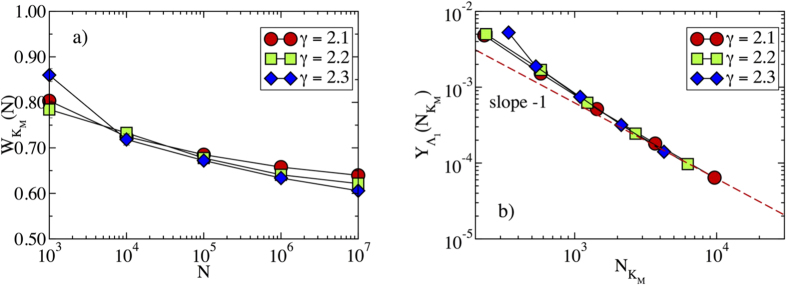
(**a**) Total weight 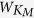 of the PEV on the nodes of the max *K*-core in synthetic networks as a function of size *N*. (**b**) Inverse participation ratio as a function of the size of the max *K*-core 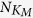. The dashed line represents a power-law behavior 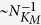. We can see the asymptotic behavior 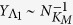, valid for large network sizes.

We can see this effective localization on the maximum *K*-core in different ways. In the first place, in Fig. 2(b,main) we plot the squared components 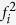 of the PEV for all vertices against their corresponding *K*-core index. From this Figure we conclude that all nodes with the largest *f*_*i*_ components belong to the max *K*-core. The size of this max *K*-core, 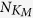, grows sublinearly as a function of the network size as 
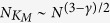
 27. However, despite this sublinear growth, a finite fraction of the total PEV weight is concentrated on this subset. We check this fact in Fig. 3(a): the total weight of the nodes in the max *K*-core,


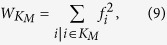


tends to a constant in the limit of large network size, implying that more than half of the weight of the normalized PEV resides over the max *K*-core. Finally, the size dependence of the max *K*-core translates, from Eq. (4) in an IPR scaling as 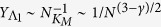, in agreement with the result obtained from the degree dependence of the PEV components, *f*_*i*_ ~ *q*_*i*_, see Eq. (8). The relation between IPR and max *K*-core size is satisfactorily checked in Fig. 3(b), where we observe it to be valid for large network sizes.

**Figure 4:**
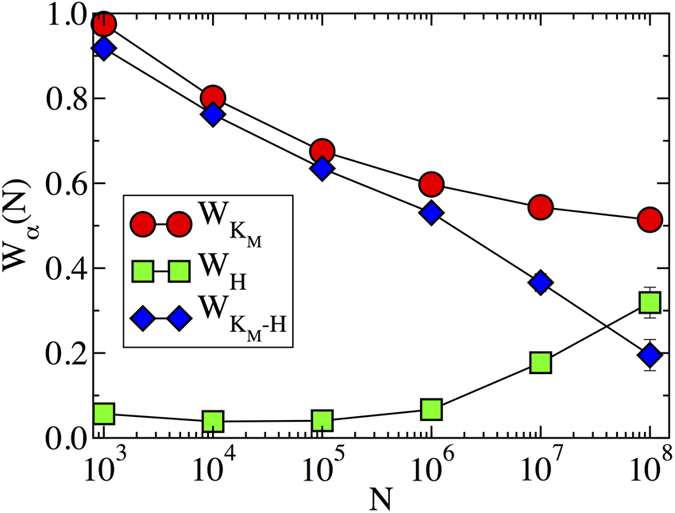
Weight of the PEV as a function of the network size in power-law networks with degree exponent *γ* = 2.8. The different functions correspond to: total weight of the nodes in max *K*-core, 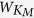; total weight in the hub, *W*_*H*_; total weight in the max *K*-core, subtracting the hub, 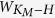.

For *γ* > 5/2, instead, Fig. 2(b,inset) confirms the localization of the PEV around the hub^14^,^16^, displaying a disproportionately large component on the node with the largest degree. Notice that, irrespective of the value of *γ*, with high probability the hub *belongs* to the max *K*-core. What changes in the two cases is that for *γ* > 5/2 the hub alone carries a finite fraction of the normalization weight 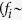 (1)) while for *γ* < 5/2 it carries a vanishing fraction, and all nodes of the max *K*-core must be considered to have a finite weight 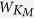. The behavior for *γ* > 3 is clearly evident from Fig. 2(b,inset). In the case 5/2 < *γ* < 3, the accumulation of a finite weight on the hub takes place for sufficiently large *N*. This effect is observed in Fig. 4, were we plot the total weight 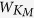 of the nodes in the max *K*-core, Eq. (9), the total weight in the hub, *W*_*H*_, and the total weight in the max *K*-core, subtracting the hub, 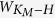. As we can observe from this Figure, the weight at the hub is small for network sizes *N* < 10^6^, but it then starts to increase, to finally take over, for large network sizes *N* > 10^7^.

### The non-backtracking centrality

The observations presented here, together with the arguments provided by Martin et *al.*^16^, hint that the EC is problematic as a useful measure of centrality. For large values of *γ*, it is affected by an exceedingly strong localization on the hub, arising as a purely topological artifact: the hub is central because its neighbors are central, but those in turn are central only because of the hub. For small values of *γ*, on the other hand, the observed relation *f*_*i*_ ~ *q*_*i*_ indicates that the eigenvector centrality provides essentially the same information as the degree centrality. As an attempt to correct the flaws of the EC, Martin et *al.*^16^ propose a modified centrality measure, the non-backtracking centrality (NBTC), which is computed in terms of the non-backtracking matrix. The Hashimoto, or non-backtracking matrix (NBT)^16^,^30^,^31^, is defined as follows: an initially undirected network is converted into a directed one by transforming each undirected edge into a pair of directed edges, each pointing in opposite directions. If the initial undirected network has *E* edges, the NBT matrix is a 2*E* × 2*E* matrix with rows and columns corresponding to directed edges *i* → *j* with value *B*_*i*→*j*,*l*→*m*_ = *δ*_*i*,*m*_(1 − *δ*_*j*,*l*_), *δ*_*i*,*j*_ being the Kronecker symbol. The components of the principal eigenvector of the NBT matrix, *f*_*i*→*j*_ measure the centrality of vertex *i* disregarding the contribution of vertex *j*. The NBT centrality of vertex *j* is given by the sum of these contributions for all neighbors of *j*: 
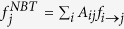
. The elements of the NBT matrix count the number of non-backtracking walks in a graph and hence remove self-feedback in the calculation of node centrality, thus eliminating in principle the artificial topological enhancement of the hub’s centrality.

**Figure 5:**
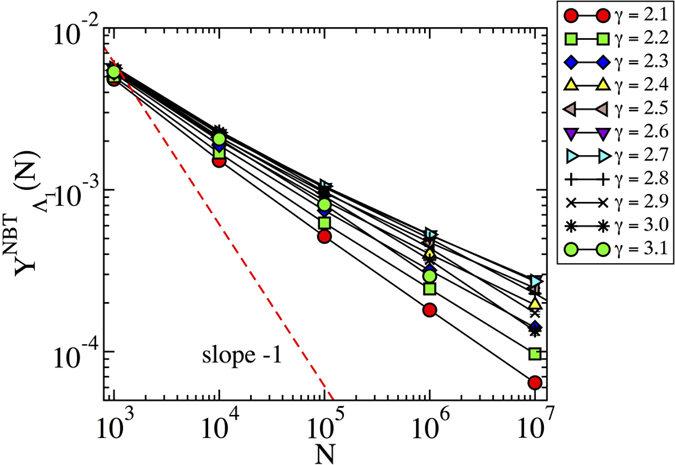
Inverse participation ratio as a function of the network size *N* for the NBTC for power-law synthetic networks with different degree exponents *γ*. The dashed line has slope −1 indicating delocalization.

**Figure 6:**
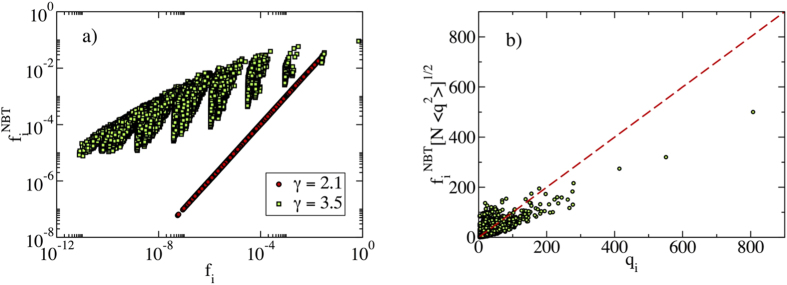
(**a**) Scatter plot of the NBTC centralities 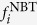 as a function of the corresponding components of the PEV of the adjacency matrix *f*_*i*_, in synthetic uncorrelated networks with a power-law degree distribution. Network size *N* = 10^6^. (**b**) Rescaled scatter plot of the NBTC centralities 
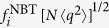
 as a function of *q*_*i*_ for a synthetic network with *γ* = 3.5 and size *N* = 10^6^.

As Fig. 5 shows, however, the NBTC is not free from localization effects: For all values of *γ* the NBTC is not delocalized, i.e. 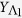 does not decrease as 1/*N* when increasing *N*. This fact can be understood for *γ* < 5/2 in view of the previous results. The adjacency matrix PEV is localized on the max *K*-core, which features many mutual interconnections: the centrality of a node is only weakly affected by self-feedback, and removing the contribution of backtracking paths has therefore little effect. This is confirmed by the scatter plot of the NBTC values 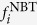 as a function of the corresponding components *f*_*i*_ of the adjacency matrix PEV, computed for the same synthetic networks, Fig. 6(a). For *γ* < 5/2 the two quantities are very strongly correlated. For *γ* > 5/2 instead, Fig. 6(a) shows that the NBT centrality is truly different and uncorrelated from the adjacency matrix EC. However, as Fig. 5 shows, the NBT IPR, computed from the components of the NBTC, decreases with the system size *N* more slowly than *N*^−1^. This is indicative that also in this case a localization occurs on a mesoscopic subset, whose size grows sublinearly. Figure 6(b) shows that this localization is not due to a strong correlation between the NBT centrality and the degree of nodes, contrary to what happens for the EC for *γ* < 5/2.

### Eigenvector localization in real networks

For real networks, which have fixed size and do not allow for a finite size scaling analysis, localization is necessarily a more blurred concept. The value of 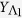 gauges how localized the PEV is, but it does not permit to unambiguously declare a network localized or not. However, also in this case it is possible to detect, as in synthetic networks, the existence of different localization modes. We consider here several real complex networks exhibiting large variations in size, heterogeneity and degree correlations (see Methods and Supplemental Material, SM, for details).

**Figure 7:**
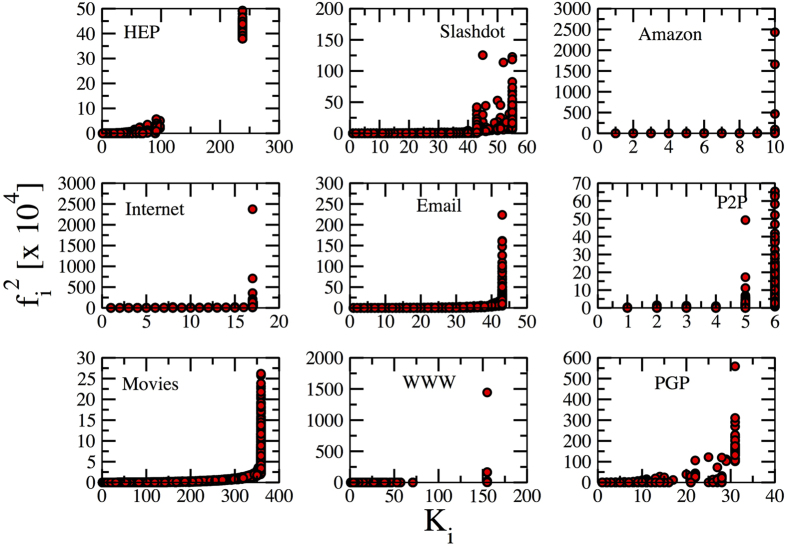
Scatter plot of squared PEV components of the adjacency matrix of the real-world networks as a function of the *K*-core index.

The linear relation between *f*_*i*_ and the degree *q*_*i*_ is not fulfilled in real networks (see Supplementary Figure SF-1), probably due to the presence of nontrivial degree correlations (see SM) which are absent in the synthetic networks. The effective localization on the max-*K* core is however still present in some cases. In Fig. 7 we plot for these networks the squared PEV component 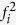 as a function of the *K*-core index. In some cases (HEP, Movies) all nodes in the max *K*-core have a comparable and large EC (as in synthetic networks for *γ* < 5/2), suggesting localization on the max *K*-core. In other cases (Internet, Amazon) one or a few nodes have a disproportionately large value of 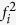, hinting at a localization around hubs, as in synthetic networks for large *γ*.

To clarify the phenomenology we report in Table 1 for each of the real-world networks the values of the leading eigenvalue, and the factors 〈*q*^2^〉/〈*q*〉 and 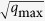. The analysis here is complicated by the presence of degree correlations (see SM), which invalidate the direct connection^11^ between Λ_1_ and the largest between 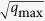 and 〈*q*^2^〉/〈*q*〉^14^. However, in some cases (Internet, Amazon) the leading eigenvalue is much closer to 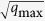 than to 〈*q*^2^〉/〈*q*〉: This suggests a localization around the hub and matches well with Fig. 7. In others the opposite is true: Λ_1_ is very far from 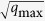 and relatively close to 〈*q*^2^〉/〈*q*〉, hinting at a localization on the max *K*-core, again in agreement with Fig. 7. In other cases (P2P, WWW), values are so close that no conclusion can be drawn.

**Figure 8:**
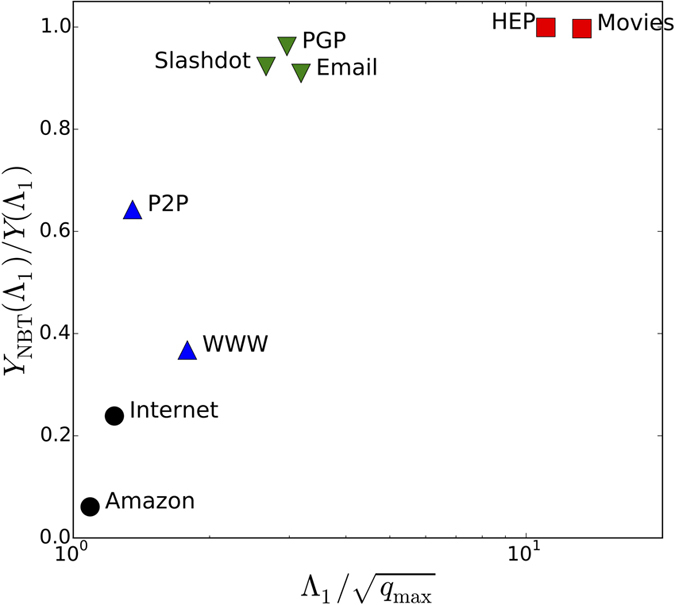
Ratio between the NBTC IPR and the IPR of the adjacency matrix as a function of the ratio between the largest eigenvalue and the square root of the maximum degree, for the real networks considered. The symbol codes are: square for localization on the max *K*-core; circle for localization on the hub; triangle up for networks in which 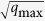 is very close to 〈*q*^2^〉/〈*q*〉, so no conclusion can be drawn; triangle down for the rest of networks.

A further confirmation of this picture is provided by the analysis of the NBT centrality. When localization occurs on hubs one expects the elimination of backtracking paths to have a strong impact, as self-feedback effects are tamed. In this case we expect the ratio between the IPR for the NBTC and the IPR for the adjacency matrix to be small. On the contrary, when the localization occurs on the max *K*-core, passing from the adjacency to the NBT matrix would not lead to a big change and we expect the ratio to be close to 1. Table 1 confirms this expectation: the IPR ratio is small when the leading eigenvalue Λ_1_ is essentially given by 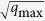 (localization on hubs) while it is close to 1 when Λ_1_ is closer to the 〈*q*^2^〉/〈*q*〉 factor (localization on the max *K*-core). A visual representation of these results is provided in Fig. 8, where we plot the IPR ratio as a function of the ratio between Λ_1_ and 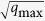. As we can see, networks in which the PEV is localized in the max *K*-core are situated in the upper right corner of the panel, while the lower left corner shows the networks with localization occurring on the hubs.

## Discussion

The properties of the principal eigenvector (PEV), and associated largest eigenvalue, of the adjacency matrix defining a network have a notable relevance as characterizing several features of its structure and its effects on the behavior of dynamical processes running on top of it. Most important among these features is the role of the components of the PEV as a measure of a node’s importance, the so-called eigenvector centrality. One of the properties of the PEV that has recently attracted the interest of the statistical physics community is its localization. In the case of networks with a power-law degree distribution *P*(*q*) ~ *q*^−*γ*^, initial research on this subject^14^,^16^ suggested that, for *γ* > 5/2, the PEV is localized on the nodes with largest degree. On the other hand, for *γ* < 5/2, the PEV should be delocalized.

In this paper we have shown that eigenvector localization in heterogeneous networks is described by a more complex picture. Thus, we present evidence that for all power-law distributed networks the PEV is always localized to some extent. In the case of synthetic power-law distributed networks, we observe that, while for mildly heterogeneous networks with *γ* > 5/2 the PEV is indeed localized on the nodes with maximum degree (the hubs), in the case of high heterogeneity, with *γ* < 5/2, the PEV shows a peculiar form of localization, its components *f*_*i*_ being proportional to the node’s degree, *f*_*i*_ ~ *q*_*i*_. This particular proportionality induces an effective localization on the maximum *K*-core of the network, defined as the core of maximum index in a *K*-core decomposition. This max *K*-core concentrates a finite fraction of the normalized weight of the PEV, despite the fact that the size of the max *K*-core is sublinear with the network size. In the case of real world networks, the elucidation of the PEV localization is not so clearcut. We however provide evidence for an analogous scenario as that observed in synthetic networks, where the nature of the localization of the PEV is ruled by its associated largest eigenvalue Λ_1_: When Λ_1_ is close to the mean-field value 〈*q*^2^〉/〈*q*〉, localization on the max *K*-core is expected. On the other hand, when the largest eigenvalue is close to 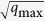, localization takes place on the hubs.

The results presented here give a new perspective on complex topologies from several viewpoints. Firstly, it is common knowledge that networks with *γ* > 3 are fundamentally different from those with *γ* < 3 (scale-free networks) because the divergence of the second moment of the degree distribution has a series of crucial effects. A tacit corollary is that networks with 2 < *γ* < 3 have essentially the same properties. Our paper, together with other recent results^14^, points out that networks with exponent *γ* < 5/2 are in many respects qualitatively different from those with *γ* > 5/2. Secondly, our results point out the weakness of eigenvector centrality as a measure of centrality for power-law networks. Indeed, for *γ* < 5/2, eigenvector centrality does not provide more information than degree centrality, while for *γ* > 5/2 the eigenvector localization on the hubs arises as a purely topological artifact. Alternative measures of centrality, based on the Hashimoto non-backtracking matrix^16^,^30^,^31^ are also not free from localization effects. Finally, from a dynamical point of view, largest eigenvalues and the associated eigenvectors are crucially related to the properties of processes on networks^7^,^14^,^32^ and their localization effects should be taken properly into account when developing theories relying on the structure of the adjacency matrix.

The localization properties described here call for a revision of our present understanding of heterogeneous topologies. Other networks properties, such as degree correlations, clustering or the presence of a community structure, might play a role in the localization of the PEV. The clarification of these effects, as well as the understanding of the nature of the mesoscopic subgraph on which the NBTC is localized for *γ* > 5/2, are still open questions, calling for further scientific effort.

## Methods

### Real networks analyzed

We consider in our analysis the following real networks datasets:

- **HEP**: Collaboration network between authors of papers submitted to the High Energy Physics section of the online preprint server arXiv. Each node is a scientist. Two scientists are connected by an edge if they have coauthored a preprint^33^.
- **Slashdot**: User network of the Slashdot technology news website. Nodes represent users, which can tag each other as friends or foes. An edge represents the presence of a tagging between two users^34^.
- **Amazon**: Co-purchasing network from the online store Amazon. Nodes represent products, which are joined by edges if they are frequently purchased together^35^.
- **Internet**: Internet map at the Autonomous System level, collected at the Oregon route server. Vertices represent autonomous systems (aggregations of Internet routers under the same administrative policy), while edges represent the existence of border gateway protocol (BGP) peer connections between the corresponding autonomous systems^36^.
- **Email**: Enron email communication network. Nodes represent email addresses. An edge joins two addresses if they have exchanged at least one email^34^.
- **P2P**: Gnutella peer-to-peer file sharing network. Nodes represent hosts in the Gnutella system. An edge stands for a connection between two Gnutella hosts^33^.
- **Movies**: Network of movie actor collaborations obtained from the Internet Movie Database (IMDB). Each vertex represents an actor. Two actors are joined by an edge if they have co-starred at least one movie^37^.
- **WWW**: Notre Dame web graph. Nodes represent web pages from University of Notre Dame. Edges indicate the presence of a hyperlink pointing from one page to another^38^.
- **PGP**: Social network defined by the users of the pretty-good-privacy (PGP) encryption algorithm for secure information exchange. Vertices represent users of the PGP algorithm. An edge between two vertices indicates that each user has signed the encryption key of the other^39^.

Some of this networks are actually directed. We have symmetrized them, rendering them undirected, to perform our analyses.

## Additional Information

**How to cite this article**: Pastor-Satorras, R. and Castellano, C. Distinct types of eigenvector localization in networks. *Sci. Rep.* **6**, 18847; doi: 10.1038/srep18847 (2016).

## Supplementary Material

Supplementary Information

## Figures and Tables

**Table 1:** Relevant metrics for the various real-world networks with and the measured value of the IPR ratio between 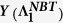 and *Y* (Λ_1_).

Network	*N*	〈*q*〉	〈*q*^2^〉/〈*q*〉	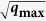	Λ_1_	*Y*(Λ_1_)	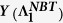	IPR Ratio
HEP	12006	19.74	129.94	22.16	244.93	0.003890	0.003887	0.9993
Slashdot	82168	12.27	149.71	50.52	134.63	0.002174	0.002006	0.9228
Amazon	403394	12.11	30.55	52.46	57.15	0.089122	0.005423	0.0608
Internet	10790	4.16	259.46	48.34	59.58	0.066138	0.015783	0.2386
Email	36692	10.02	140.08	37.19	118.42	0.003790	0.003446	0.9091
P2P	62586	4.73	11.60	9.75	13.18	0.000921	0.000592	0.6429
Movies	81860	89.53	594.92	61.55	817.36	0.000640	0.000638	0.9966
WWW	325729	6.69	280.68	103.54	184.93	0.022726	0.008357	0.3677
PGP	10680	4.55	18.88	14.32	42.44	0.016622	0.015989	0.9619

Size and other information on the networks are provided in the Supplementary Information.
