# Supplementary material for: Distinct types of eigenvector localization in networks
Source: Sci Rep. 2016 Jan 12;6:18847. doi: 10.1038/srep18847 (PMC4709588; doi:10.1038/srep18847)
Supplement: Supplementary Information [file srep18847-s1.pdf]

Distinct types of eigenvector localization in networks: Supplemental Material

Romualdo Pastor-Satorras[1] and Claudio Castellano[2,3]

[1]Departament de Física i Enginyeria Nuclear, Universitat Politècnica de Catalunya, Campus Nord B4, 08034 Barcelona, Spain

[2]Istituto dei Sistemi Complessi (ISC-CNR), Via dei Taurini 19, I-00185 Roma, Italy

[3]Dipartimento di Fisica, "Sapienza" Università di Roma, P.le A. Moro 2, I-00185 Roma, Italy

1 Topological properties of the real networks considered

In Table SM-1 we present a summary of the basic statistical properties of the real networks analyzed in this paper (see Methods for a description). In Figure SM-1 we plot the corresponding degree distribution for each network. As we can see, all them have a long tailed degree distribution, with varying values of the degree exponent in a fit to a pure power-law form, ranging from 1.7 to 4.6 (see Table SM-1).

Network	γ	r	$q_{\max}$	K_M	N_{K_M}
HEP	1.8	0.63228	491	238	239
Slashdot	1.7	-0.07383	2552	55	134
Amazon	3.1	-0.01765	2752	10	32886
Internet	2.2	-0.19380	2337	17	34
Email	1.9	-0.11076	1383	43	275
P2P	4.6	-0.09256	95	6	1004
Movies	1.8	0.20595	3789	359	1125
WWW	2.6	-0.05344	10721	155	1367
PGP	2.0	0.23821	205	31	41

Table SM-1: Topological properties of the real networks considered: γ effective degree exponent in a fit of the degree distribution to the power-law form $P(q) \sim q^{-\gamma}$; r Pearson degree correlation coefficient, measuring degree correlations [1]; $q_{\max}$ maximum degree; K_M maximum K-core index; N_{K_M} size of the maximum K-core [2].

Real networks show different levels of degree correlations as measured from the average degree of the nearest neighbors of the vertices of degree q, $q_{\rm nn}(q)$ [3](see Figure SM-2) and the Pearson degree correlation r [1](see Table SM-1).

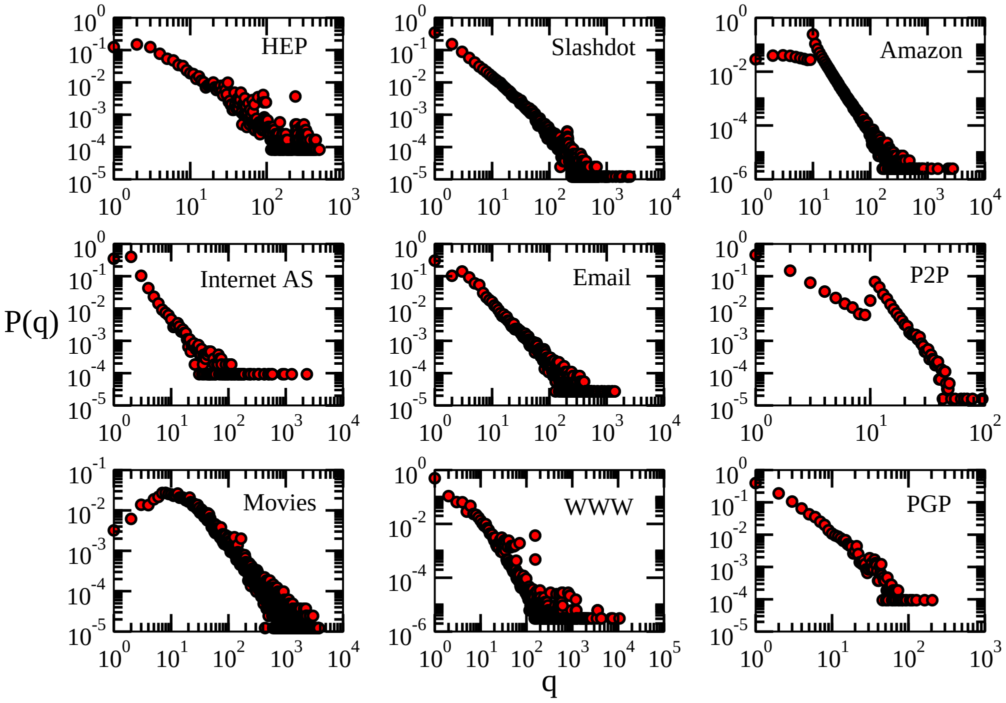

Figure SM-1: Degree distribution of the real networks considered.

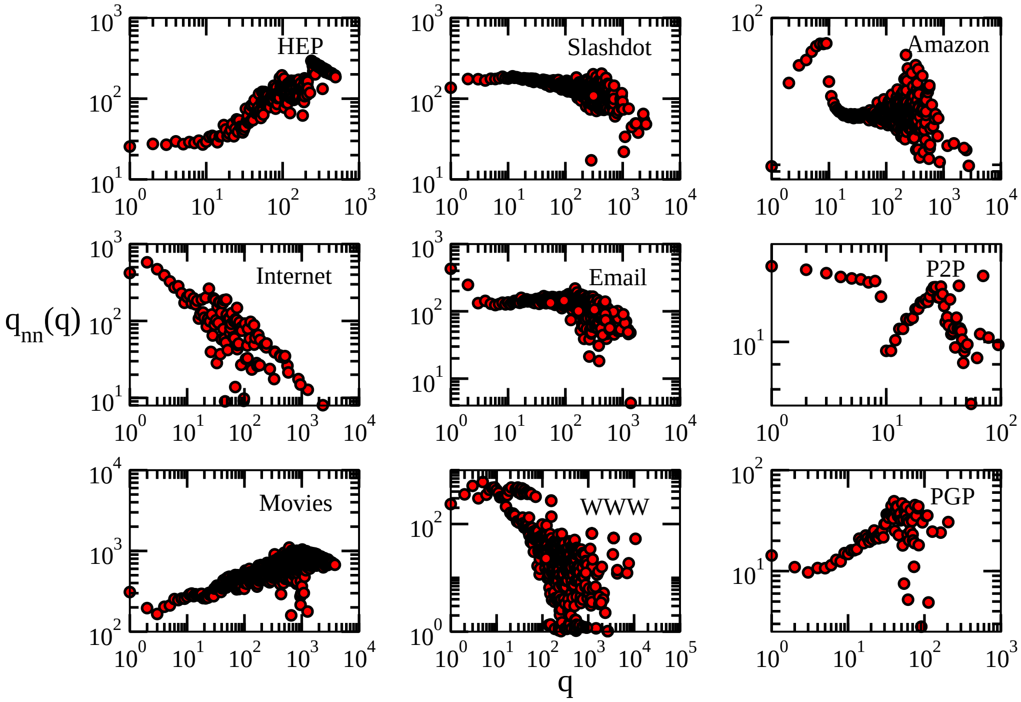

Figure SM-2: Average degree of the nearest neighbors as a function of the degree q of the real networks considered.

Supplementary Figures

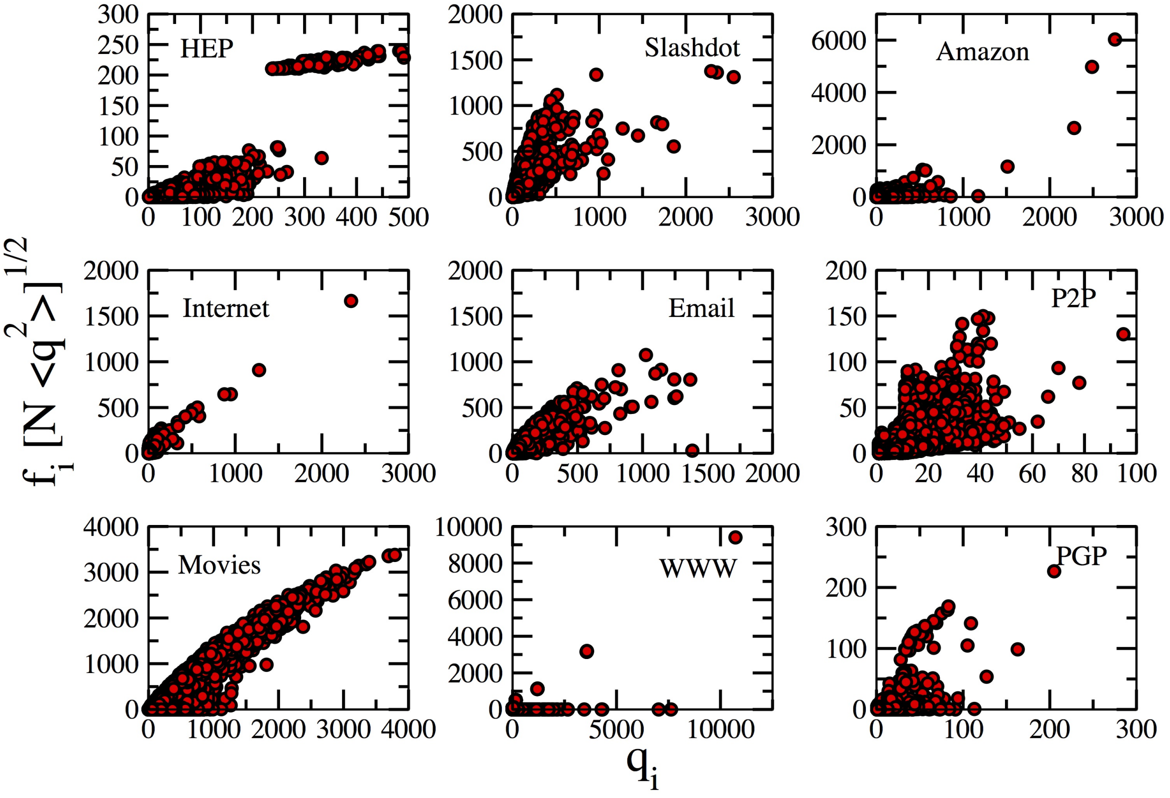

Figure SF-1: Rescaled scatter plot of $f_i[N\langle q^2\rangle]^{1/2}$ as a function of q_i for real-world networks.

References

[1] Newman, M. E. J. Assortative mixing in networks. *Phys. Rev. Lett.* **89**, 208701 (2002).

[2] Seidman, S. B. Network structure and minimum degree. *Social Networks* **5**, 269 –287 (1983).

[3] Pastor-Satorras, R., Vázquez, A. & Vespignani, A. Dynamical and correlation properties of the Internet. *Phys. Rev. Lett.* **87**, 258701 (2001).
